# ATM-depletion in breast cancer cells confers sensitivity to PARP inhibition

**DOI:** 10.1186/1756-9966-32-95

**Published:** 2013-11-19

**Authors:** Maria Saveria Gilardini Montani, Andrea Prodosmo, Venturina Stagni, Dania Merli, Laura Monteonofrio, Veronica Gatti, Maria Pia Gentileschi, Daniela Barilà, Silvia Soddu

**Affiliations:** 1 Department of Ecological and Biological Sciences, Tuscia University, Largo dell’Università, 01100 Viterbo, Italy; 2 Experimental Oncology, Regina Elena National Cancer Institute, Via Elio Chianesi, 53-00144 Rome, Italy; 3 Laboratory of Cell Signaling, IRCCS-Fondazione Santa Lucia, Via Ardeatina 306, 00179 Rome, Italy; 4 Department of Biology, University of Rome “Tor Vergata”, Via della Ricerca Scientifica, 00133 Rome, Italy

**Keywords:** Breast cancer, ATM, PARP inhibitors, Olaparib, Iniparib

## Abstract

**Background:**

Mutations in the DNA damage response (DDR) factors, breast cancer 1 (BRCA1) and BRCA2, sensitize tumor cells to poly(ADP-ribose) polymerase (PARP) inhibitors. The ataxia telangiectasia mutated (ATM) kinase is a key DDR protein whose heterozygous germline mutation is a moderate–risk factor for developing breast cancer. In this study, we examined whether ATM inactivation in breast cancer cell lines confers sensitivity to PARP inhibitors.

**Methods:**

Wild-type BRCA1/2 breast cancer cells (*i.e.*, MCF-7 and ZR-75-1 lines) were genetically manipulated to downregulate ATM expression then assayed for cytostaticity/cytotoxicity upon treatment with PARP inhibitors, olaparib and iniparib.

**Results:**

When ATM-depleted cells and their relative controls were treated with olaparib (a competitive PARP-1/2 inhibitor) and iniparib (a molecule originally described as a covalent PARP-1 inhibitor) a different response to the two compounds was observed. ATM-depletion sensitized both MCF-7 and ZR-75-1 cells to olaparib-treatment, as assessed by short and long survival assays and cell cycle profiles. In contrast, iniparib induced only a mild, ATM-dependent cytostatic effect in MCF-7 cells whereas ZR-75-1 cells were sensitive to this drug, independently of ATM inactivation. These latest results might be explained by recent observations indicating that iniparib acts with mechanisms other than PARP inhibition.

**Conclusions:**

These data indicate that ATM-depletion can sensitize breast cancer cells to PARP inhibition, suggesting a potential in the treatment of breast cancers low in ATM protein expression/activity, such as those arising in mutant ATM heterozygous carriers.

## Background

In the past few years, much effort has been made towards identifying chemotherapeutic compounds targeting the core components of DDR and repair pathways, which are frequently altered in tumor cells. The goal for these new anti cancer strategies would be to take advantage of the cancer cell defects in repairing their own DNA and use it as an Achille’s heel to enhance therapeutic indices, with limited normal tissue toxicity. Among these new compounds, PARP inhibitors have been shown to be highly lethal to tumor cells with deficiencies in DDR factors such as BRCA1 or BRCA2 [1,2]. The mechanism underlining this approach is based on the concept of synthetic lethality first described in the fruit fly *Drosophila *[3,4] and subsequently translated into an efficient method to design novel anticancer drugs [5,6]. Synthetic lethality centers on targeting two separate molecular pathways that are nonlethal when disrupted individually, but are lethal when inhibited simultaneously [7].

In the case of PARP inhibitors and BRCA1/2 mutations, the two molecular pathways whose concomitant inactivation promotes a synthetic lethal relationship are the basic excision repair (BER), responsible for the repair of single-strand DNA breaks (SSBs), and the homologous recombination (HR), that repairs double strand DNA breaks (DSBs). In particular, BER inactivation by PARP inhibitors induces SSBs that during DNA replication cause lethal breaks in both DNA strands. In normal cells, the latter breaks are repaired by HR, but in tumor cells in which HR is defective, such as in the presence of BRCA1/2 mutations, DSBs are not repaired and their accumulation causes cell death [1,2]. These original observations have led to PARP inhibitors entering subsequent phase II clinical trials in breast and ovarian cancer patients, with or without BRCA mutations [8-10]. At present, the data from clinical studies are not as favorable as promised by the preliminary results [11,12]. Though there might be various causes explaining the clinical performance of the different PARP inhibitors, one of the challenging issues remains on how to identify those patients most receptive to these treatments [13].

Deficiency in several DDR factors other than BRCA1/2 belonging, directly or indirectly, to the HR repair pathway have been shown to sensitize tumor cells to PARP inhibition [14] and synthetic lethal-siRNA screens have identified ATM among the genes whose depletion might mediate the sensitivity to PARP inhibitors [15]. Recently, ATM-deficient mantle cell lymphoma, chronic lymphocytic leukemia, and T-prolymphocytic leukemia have been shown to be more sensitive to PARP inhibitors than ATM-proficient cells [16,17] suggesting that ATM mutation/inactivation might predict responses of individual tumors to PARP inhibitors.

ATM is one of the key DNA damage sensors that have a critical role in contributing to DDR by regulating cell cycle checkpoints, DNA repair machinery, replication forks, and telomeres [18,19]. Homozygous mutations of ATM are responsible for ataxia-telangiectasia (A-T), a rare autosomal recessive disease mainly characterized by progressive degeneration in the cerebellum, immunodeficiency, radiosensitivity, and cancer predisposition [20,21]. Although A-T heterozygotes are usually asymptomatic and, overall considered healthy carriers, a link between single copy ATM mutations and a two to five fold risk of breast cancer has been established [22]. Recently, we have developed a straightforward, rapid, and inexpensive test to unambiguously diagnose A-T heterozygotes that would allow an easy recognition of breast cancer patients carrying monoallelic ATM germline mutations [23].

In the current studies, we assessed whether ATM depletion by RNA interference sensitize cells from breast cancer lines to PARP inhibitors. As ATM mutations and loss of ATM expression can be found in hereditary and sporadic breast cancers and A-T heterozygotes can be diagnosed [23], we hypothesized that such data might be useful in extending the molecular predictors required for selecting patients responsive to PARP inhibition.

## Materials and methods

### Cell culture and reagents

Human breast cancer cell lines, MCF-7 and ZR-75-1, and their transfected-derivatives were maintained in DMEM-Glutamax and RPMI-Glutamax, respectively, supplemented with 10% fetal bovine serum, 100 U/ml penicillin, and 100 U/ml streptomycin (all from Invitrogen). All cell lines were maintained in a 5% CO_2_ atmosphere at 37°C. Cells were passaged once every 3–5 days (~90% confluence) and all experiments were performed within the first 10 passages from transfection. For drug treatment, doxorubicin (Sigma) and PARP inhibitors, olaparib and iniparib (Selleckchem), were prepared as stock solution in water or DMSO, respectively, aliquot and stored at -80°C until use.

### Stable knockdown of ATM in cells of breast cancer lines

Stable interference was obtained by retroviral-mediated expression of short-hairpin RNA (shRNA) using pRETRO-Super vector. Retroviruses were produced in HEK 293 T cells by cotransfecting pRETRO-Super together with plasmids encoding for gag-pol and VSV-G proteins. Viral supernatant was collected 48 hrs post-transfection, filtered through a 0.45 μm pore size filter and added to the cells in the presence of 2 μg/ml polybrene. After 48 hrs from infection, stable polyclonal populations of control and ATM-depleted cells were obtained by selection for two weeks with 2 μg/ml puromycin (Sigma).

The shATM construct (#1 position 912) in pRETRO-Super, generously provided by Y. Lerenthal and Y. Shiloh, has the following sequence: 5′-GAC TTT GGC TGT CAA CTT TCG-3′ [24]. Control shRNA, siR5, has the following sequence: 5′-GGA TAT CCC TCT AGA TTA-3′. Neither the ATM-targeting shRNA nor the control sequences have any homology with other human gene as tested by BLAST (http://blast.ncbi.nlm.nih.gov/Blast.cgi).

### Western blotting

Total cell extracts were prepared in lysis buffer [50 mM Tris–HCl pH 8, 300 mM NaCl, 1 mM EDTA, 0.5% sodium deoxycholate, 0.1% SDS, 1% Nonidet-P40, 1 mM EDTA] supplemented with protease-inhibitor mix (Roche), resolved on precast NuPAGE 4-12% gels (Invitrogen), and transferred onto nitrocellulose membranes (Bio-Rad). The following antibodies were employed for immunedetection: rabbit anti-ATM (Santa Cruz), mouse anti-α-tubulin (Immunological Sciences), HRP-conjugated goat anti-mouse and anti-rabbit (Cappel). Immunoreactivity was determined using the ECL-chemiluminescence reaction (Amersham Corp) following the manufacturer’s instructions.

### Ionizing radiation (IR)

When indicated, cells were irradiated using a ^137^Cs source (IBL-437-C irradiator, CIS bio International) at a dose rate of 6.8 Gy/min.

### Citotoxicity and BrdU assays

Cells (5 × 10^4^/ml) were seeded in 96-well plates in growth medium and incubated 24 hrs at 37°C in 5% CO_2_ atmosphere. Drugs were added at the indicated concentrations and for the indicated times before incubation with reagents of XTT, WST-1, and BrdU (all from Roche Applied Science), following the manufacturer’s instructions. The absorbance at 450 nm (XTT and WST-1) or at 370 nm (BrdU) were measured by the microplate reader Infinite F200 (Tecan). Each experiment was performed in triplicate. The survival fraction for a given dose was calculated as the plating efficiencies for that dose divided by the plating efficiencies of solvent-treated cells.

### Cell cycle profiles

Treated and untreated cells (5 × 10^5^) were washed in PBS 1X and resuspended in 300 μl hypotonic fluorochrome solution [50 μg/ml propidium iodide, 0.1% sodium citrate, 0.1% Triton-X-100 (all from Sigma)] for 30 min at room temperature. DNA content was measured by a FACScan flow cytometer (Becton Dickinson).

### Colony forming assays

Cells were treated with drugs at the indicated doses for 24 hrs, then plated at low density in 60 mm Petri dishes and grown for twelve days in the absence of drugs. Surviving colonies were fixed and stained with Cristal Violet (0.5% in methanol) (Sigma), air-dried, and counted.

### Statistics

The Wilcoxon test for paired samples has been used for repeated measurements. A p-value less than 0.10 (*) and less than 0.05 (**) were considered statistical significant.

## Results and discussion

### Effects of ATM-depletion in breast cancer MCF-7 cell line

**Figure 1:**
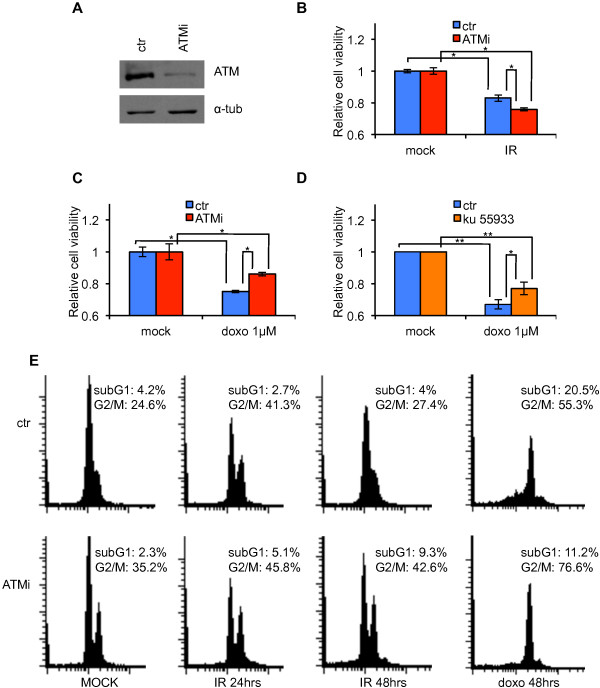
**MCF-7 transduction with shATM-carrying vectors elicits a phenotype compatible with ATM defective cells. (A)** MCF-7 cells were transfected with shATM-carrying vector (MCF7-ATMi) and its siR5 negative control (MCF7-ctr). ATM protein levels in MCF-7-ATMi and MCF-7-ctr cells were analyzed by Western blot. α-tubulin was used as an internal control. **B-D** Cell viability of MCF7-ATMi and MCF7-ctr cells upon treatment with IR **(B)** and doxorubicin **(C)**. **(D)** MCF7-ctr cells were pre-treated with ATM inhibitor KU 55933 or its solvent before addition of doxorubicin as in **(C)**. Data are represented as mean ± standard deviation (SD). **(E)** Flow cytometry analysis of cell-cycle distribution of MCF7-ATMi and MCF7-ctr cells upon treatment with IR and doxorubicin at indicated times. Asterisks indicate statistical significant difference (*P < 0.1; **P < 0.05).

To assess the influence of ATM in breast cancer susceptibility to PARP inhibitors, we genetically repressed ATM expression by RNA interference in MCF-7 cells. We chose the MCF-7 breast cancer cell line because it is ER positive, HER2 negative, and wild-type for the *BRCA1*, *BRCA2*, and *TP53* genes [25], features we observed in breast tumors arising in our A-T heterozygotes [23]. Stable interference of ATM was obtained by MCF-7 transfection with shATM-carrying vectors (MCF7-ATMi) and its siR5 negative control (MCF7-ctr) (see Materials and methods). Stable-transfected cells were selected in the presence of puromycin for ten days and maintained as polyclonal populations. As shown in Figure 1A, a strong repression of ATM expression was obtained in the MCF7-ATMi cells compared to the MCF7-ctr ones. To verify whether ATM-depletion has a functional impact on MCF-7 cells, we assessed the sensitivity of ATM-depleted and control cells to IR and doxorubicin treatment, that are known to induce different outcomes in ATM proficient and defective cells. In particular, radiosensitivity is a defining feature of ATM-defective cells [26] whereas, in a wild-type p53 context, doxorubicin-resistance was shown to characterize ATM-deficient cells in vitro [27] and in breast cancer patients [28]. As shown in Figure 1B and 1C, MCF7-ATMi cells were more sensitive to IR and more resistant to doxorubicin than MCF7-ctr cells. The contribution of ATM in the latter result was confirmed in MCF-7 parental cells by KU 55933-induced ATM inactivation (Figure 1D). These results were further confirmed by evaluating the cell cycle profiles (Figure 1E). After 24 hrs from irradiation, both MCF7-ctr and MCF7-ATMi cells show the expected enrichment into the G2/M phase. After 48 hrs from irradiation, MCF7-ctr cells repair the damage and re-enter into the cell cycle; in contrast, MCF7-ATMi cells, which are known to have defects in sensing and repairing DNA double strand breaks [26], show a delay in re-entering into the cell cycle. In contrast, as expected from the data reported by Jiang and co-workers [27], the ATMi cells were more resistant to doxorubicin and a lower proportion of cells underwent cell death.

Altogether, these results show that MCF-7 transduction with shATM-carrying vectors interferes with ATM expression and elicits some aspects of a phenotype compatible with ATM-deficient cells.

### ATM-depletion sensitizes MCF-7 cells to olaparib

**Figure 2:**
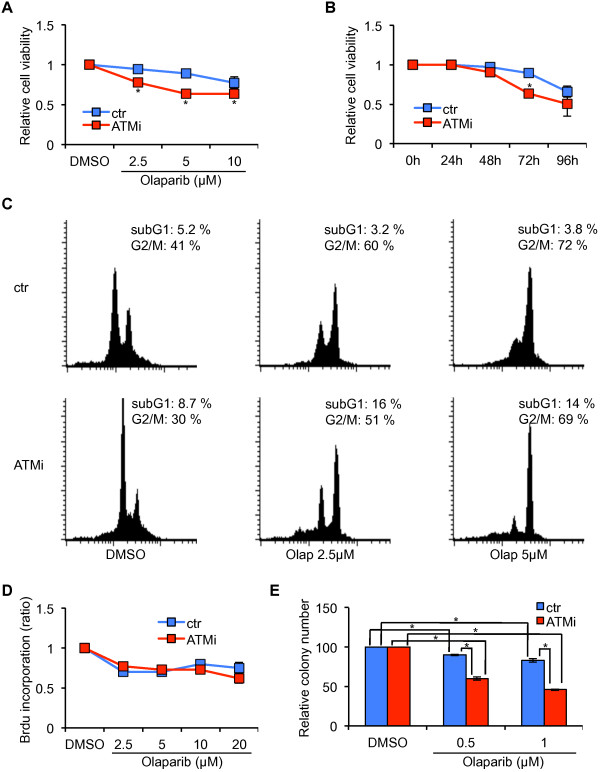
**MCF7-ATMi cells are more sensitive than MCF7-ctr cells to olaparib. A-B** MCF7-ATMi and MCF7-ctr cells were exposed to increased concentrations of olaparib for 72 hrs **(A)** or were treated with olaparib (5 μM) for up to 96 hrs **(B)**. Data are represented as mean ± SD. **(C)** Flow cytometry analysis of cell-cycle distribution of MCF7-ATMi and MCF7-ctr cells treated with the indicated concentrations with olaparib for 48 hrs. **(D)** DNA synthesis was measured by BrdU incorporation assay 48 hrs after olaparib treatment. **(E)** Quantitative analyses of colony formation. The numbers of DMSO-resistant colonies in MCF7-ATMi and MCF7-ctr cells were set to 100, while olaparib treated cel1s were presented as mean ± SD. Asterisks indicate statistical significant difference (*P < 0.1).

To evaluate whether ATM-depletion modifies MCF-7 response to PARP inhibitors, we first used olaparib (AZD2281, Ku-0059436), an orally bioavailable compound whose effectiveness in BRCA1/2 mutated breast and ovarian cancers was studied in phase II clinical trials and, for ovarian cancers is under further evaluation in phase III clinical studies [12]. MCF7-ATMi and MCF7-ctr cells were incubated with increasing concentrations of olaparib or its solvent (DMSO) for 72 hrs and their viability assessed by XTT or WST-1, with comparable results. As shown in Figure 2A, ATM-depleted cells were mildly but significantly more sensitive than MCF7-ctr cells to olaparib. However, MCF7-ctr cells, as well as the parental MCF-7 cells (data not shown) were not completely resistant to olaparib and their viability declined with time (Figure 2B) and at the highest doses we employed (Figure 2A, 10 μM dose).

To further characterize the effect induced by olaparib, MCF7-ATMi and MCF7-ctr cells were treated for 48 hrs with 2.5 and 5 μM olaparib and their DNA content assessed by propidium iodide staining and FACS analysis. Consistently with the viability assays described above, cell death, measured by the appearance of hypodiploid cells, was detected only in the olaparib-treated MCF7-ATMi cells (Figure 2C). However, both ATM-depleted and control MCF-7 cells arrested in the G_2_/M phase of the cell cycle, in a dose-dependent manner, as previously described [2]. The similarity in the cell cycle behavior between MCF7-ATMi and MCF7-ctr cells after olaparib treatment was confirmed by BrdU assay that showed a comparable reduction in the two cell populations (Figure 2D). These data indicate that MCF-7 sensitivity to olaparib is increased by ATM-depletion, but these cells are partially responsive to this compound, as also recently reported by others [29].

Next, we verified the long-term effect of olaparib by performing colony formation assays. MCF7-ATMi and MCF7-ctr cells were treated for 24 hrs with 0.5 and 1 μM olaparib, then plated at low density and grown for twelve days in the absence of drug. As shown in Figure 2E, a significant reduction in the colony forming capacity was observed in the ATM-depleted cells compared to the controls. Consistent with the results described above, a mild reduction in colony formation was also observed in the olaparib-treated MCF7-ctr cells compared with their DMSO-treated controls (Figure 2E, blue columns).

Overall, these data indicate that ATM-depletion increases sensitivity to olaparib in breast cancer MCF-7 cells; however, factors other than ATM might contribute to the response of this cell line to this PARP-inhibitor.

### ATM-depletion sensitizes MCF-7 cells to iniparib

**Figure 3:**
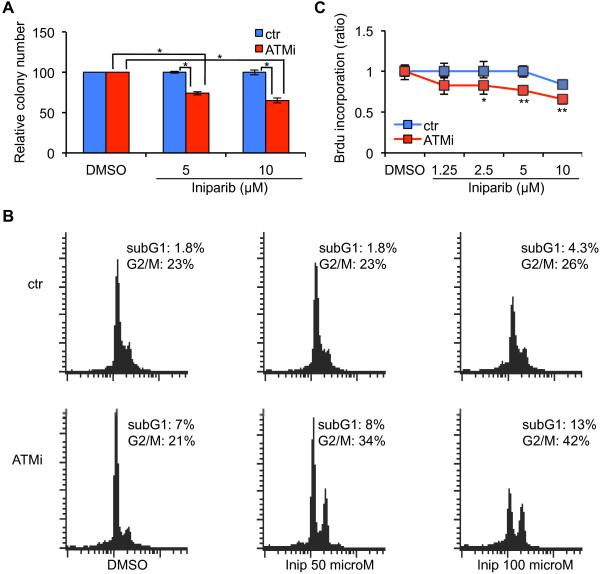
**MCF7-ATMi cells are more sensitive than MCF7-ctr cells to iniparib. (A)** Quantitative analyses of colony formation. The numbers of DMSO-resistant colonies in MCF7-ATMi and MCF7-ctr cells were set to 100, while iniparib treated cel1s were presented as mean ± SD. **(B)** Flow cytometry analysis of cell-cycle distribution of MCF7-ATMi and MCF7-ctr cells treated with the indicated concentrations of iniparib for 48 hrs. **(C)** DNA synthesis was measured by BrdU incorporation assay 48 hrs after iniparib treatment. Data are represented as mean ± SD. Asterisks indicate statistical significant difference (*P < 0.1; **P < 0.05).

Next, we asked whether ATM-depletion can sensitize MCF-7 cells to iniparib (BSI-201, SAR240550), a compound originally described as an irreversible inhibitor of PARP-1 [30], but recently shown to act as a nonselective modifier of cysteine-containing proteins [31,32]. MCF7-ATMi and MCF7-ctr cells were treated with iniparib or its solvent, DMSO, and analyzed for colony formation capacity, DNA content by FACS analysis, and BrdU assay. As shown in Figure 3A, ATM-depletion reduced the ability of MCF-7 cells to produce colonies after iniparib-treatment while no effect was observed in MCF7-ctr cells. At variance with olaparib-treatment, DNA content analysis did not reveal any significant difference between MCF7-ATMi and MCF7-ctr cells in the appearance of hypodiploid, death cells, whereas only the MCF7-ATMi population experienced an accumulation of cells in the G_2_/M phase of the cell cycle (Figure 3B). This effect on the cell cycle was confirmed by BrdU assays (Figure 3C). Together, these results suggest that ATM-depletion can also influence MCF-7 cell response to iniparib.

### ATM-depletion sensitizes ZR-75-1 breast cancer cells to olaparib but not to iniparib

**Figure 4:**
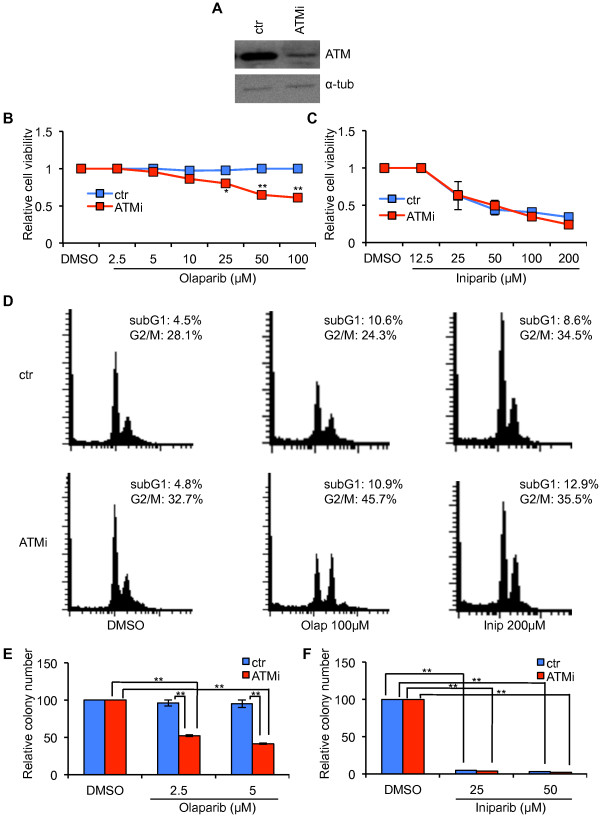
**ZR-ATMi cells are more sensitive than ZR-ctr cells to olaparib but not to iniparib. (A)** ZR-75-1 cells were transfected with shATM-carrying vector (ZR-ATMi) and its siR5 negative control (ZR-ctr). ATM protein levels in ZR-ATMi and ZR-ctr cells were analyzed by Western blot. α-tubulin was used as an internal control. **B-C** ZR-ATMi and ZR-ctr cells were exposed to increased concentrations of olaparib **(B)** or iniparib for 72 hrs **(C).** Data are represented as mean ± SD. **(D)** Flow cytometry analysis of cell-cycle distribution of ZR-ATMi and ZR-ctr cells treated with the indicated concentrations with olaparib or iniparib for 72 hrs. **E-F** Quantitative analyses of colony formation. The numbers of DMSO-resistant colonies in ZR-ATMi and ZR-ctr cells were set to 100, while olaparib **(E)** or iniparib **(F)** treated cel1s were presented as mean ± SD. Asterisks indicate statistical significant difference (*P < 0.1; **P < 0.05).

To further assess the impact of ATM-depletion in breast cancer cell response to olaparib and iniparib, we selected the ZR-75-1 line, whose cells, like the MCF-7 ones, are ER positive, HER2 negative, and wild-type for *BRCA1*/*2* and *TP53* genes [25]. Stable interference of ATM in ZR-75-1 cells was obtained as described for MCF-7 cells. Polyclonal populations, ZR-ATMi and ZR-ctr, were obtained by puromycin selection and ATM-depletion confirmed by Western blot analysis (Figure 4A). Next, dose–response viability assays were performed on ZR-ATMi and ZR-ctr cells upon incubation with olaparib, iniparib, or their solvent, DMSO. As shown in Figures 4B, ZR-ctr cells were strongly resistant to olaparib whereas their ATM-depleted counterpart became considerably sensitive and showed a partial accumulation in the G2/M phase of the cell cycle (Figure 4D). These results, confirmed by colony formation assays (Figure 4E), sustain the observations made with MCF-7 cells and support a synthetic lethal relationship between ATM-depletion and olaparib-treatment in ER positive, wild-type BRCA 1/2 breast cancer cells.

In contrast with the sensitivity induced by ATM-depletion in MCF-7 cells, when treated with iniparib, both ZR-ATMi and ZR-ctr cells showed a substantial loss of viability that was independent of ATM, as indicated by the similarity of their survival curves (Figure 4C) and cell cycle distribution (Figure 4D). These results were confirmed by the complete inhibition of colony formation induced by iniparib in ZR-75-1 cells, independent of their ATM status (Figure 4F). In addition, the different response between MCF-7 and ZR-75-1 cells to this drug suggests that ER expression and the wild-type status of BRCA1/2 and TP53 are not involved in the sensitivity to iniparib. These results might be explained by the recent observations indicating that the primary mechanism of action for iniparib is a nonselective modification of cysteine-containing proteins, rather then inhibition of PARP activity [32].

## Conclusions

In a few hematological malignancies, ATM-deficiency was shown to confer sensitivity to PARP inhibitors, indicating that ATM might be included in the DDR factors whose mutation or loss of expression confer sensitivity to this class of drugs. Based on these observations, we asked whether ATM deficiency plays a similar role in breast cancer, the solid tumor linked to ATM germline mutations. For this study, we employed two breast-cancer cell lines selected among those showing the molecular feature we recently observed in the breast tumors arising in A-T heterozygotes. In addition, we selected two compounds, olaparib and iniparib, originally described as PARP inhibitors. We show that ATM-depletion confers sensitivity to olaparib in both cell lines and a mild sensitivity to iniparib in the MCF-7 cells indicating that ATM mutation/inactivation might be consider in the selection of breast cancers responsive to PARP inhibition.

## Abbreviations

ATM: Ataxia telangiectasia mutated; BRCA1/2: Breast cancer 1/2; DDR: DNA damage response; IR: Ionizing radiation; PARP: Poly(ADP-ribose) polymerase; PBS: Phosphate buffered saline; SD: Standard deviation; shRNA: Short hairpin RNA.

## Competing interests

The authors declare that they have no competing interests.

## Authors’ contributions

MSGM and DM performed cytotoxicity and assays, clonogenicity and cell cycle profiles. AP, VS and LM performed shRNA transfection, cell selection, and western blotting. MPG and VG were responsible for cell handling. MSGM, AP, DB and SS were involved in the experimental design and conception, data collection and analysis. SS wrote the manuscript. All authors read and approved the final manuscript.
